# Who is at risk of bias? Examining dispositional differences in motivated science reception

**DOI:** 10.1177/09636625241262611

**Published:** 2024-07-31

**Authors:** Marlene Sophie Altenmüller, Laura Amelie Poppe

**Affiliations:** Ludwig-Maximilians-Universität München, Germany

**Keywords:** conspiracy mentality, motivated science reception, personality, trust in science, victim sensitivity

## Abstract

The motivated reception of science in line with one’s preexisting convictions is a well-documented, pervasive phenomenon. In two studies (*N* = 743), we investigated whether this bias might be stronger in some people than others due to dispositional differences. Building on the assumptions that motivated science reception is driven by perceived threat and suspicion and higher under perceived ambiguity and uncertainty, we focused on traits associated with such perceptions. In particular, we tested the impact of conspiracy mentality and victim sensitivity on motivated science reception (as indicated by ascriptions of researchers’ trustworthiness and evidence credibility). In addition, we explored the role of broader personality traits (generalized mistrust and ambiguity intolerance) in this context. None of the investigated dispositions modulated the motivated science reception effect. This demonstrates once again, that motivated science reception is a ubiquitous challenge for the effective dissemination of science and everyone seems to be at risk of it.

We all like to see the world in a way that fits with our preexisting beliefs. This inclination toward motivated reasoning (Kunda, 1990) does not stop at science. A wealth of research has demonstrated the pervasive phenomenon of “motivated science reception” (Rothmund et al., 2017): People perceive scientists and scientific evidence depending on their own attitudes, beliefs, and identity (e.g. Altenmüller et al., 2021; Bender et al., 2016; Greitemeyer, 2014; Hutmacher et al., 2022; Nauroth et al., 2015, 2017; Nisbet et al., 2015; Rothmund et al., 2015). For example, highly identified gamers depreciated research that reported a link between aggression and video games because it presented a threat to their social identity (Nauroth et al., 2015).

This bias might be stronger in some people than others. Especially individuals who are prone to feelings of threat and suspiciousness and dislike ambiguity might be inclined to reject scientific findings that are incongruent to their own convictions. This assumption is supported by the video game example above, demonstrating that motivated rejection of science is, at least partially, driven by perceived threat (e.g. Bender et al., 2016; Nauroth et al., 2015, 2017). Moreover, motivated science reception has been shown to be more pronounced when there is uncertainty or ambiguity associated with the communicated science (e.g. Altenmüller et al., 2021; Dieckmann et al., 2017). For example, there is growing evidence that motivated rejection of climate science (in particular, among conservatives) can be reduced by emphasizing the high scientific consensus on the issue (i.e. decreasing perceived uncertainty; Van der Linden et al., 2019; Van Stekelenburg et al., 2022).

Surprisingly, it has rarely been investigated whether interindividual differences alleviate or amplify the inclination for motivated science reception. While first evidence suggests that this bias might, in principle, be malleable, research has so far only focused on differences in particular science-related skills. For example, Hutmacher et al. (2022) showed that numeracy reduced the biased evaluation of scientific findings. Other research (Drummond and Fischhoff, 2017) suggested that scientific literacy can increase polarization, but this finding has been questioned (Fischer et al., 2022). Moving beyond skills, it seems worthwhile to take a closer look at the role of personality traits in motivated science reception.

Considering that motivated science reception has been linked to perceived threat and uncertainty, personality traits which are also associated with this appear as prime candidates as modulators of motivated science reception. Besides broader predispositions like generalized mistrust or intolerance of ambiguity, two traits seem particularly interesting in this regard: conspiracy mentality (CM) and victim sensitivity (VS).

## Conspiracy mentality

CM is a dispositional tendency to believe in conspiracies (Bruder et al., 2013; Milošević Đorđević et al., 2021). It has been suggested that conspiracy belief is driven by perceived uncertainty and loss of and need for control (Van Prooijen and Acker, 2015; Van Prooijen and Douglas, 2017; Van Prooijen and Jostmann, 2013). In line with this, CM has been linked to feelings of lacking (sociopolitical) control and power (Bruder et al., 2013), and, more generally, a worldview characterized by distrust in others and perceived threat by powerful elites (Frenken and Imhoff, 2023; Imhoff et al., 2018; Imhoff and Bruder, 2014).

Applying these insights to science reception, it is possible that scientists might be seen as such a threatening powerful elite. There is first evidence for this regarding physicists and historians (Imhoff et al., 2018; Imhoff and Bruder, 2014). More generally, a conspiracist worldview has been suggested as one of the roots of science skeptical attitudes (Hornsey, 2020). However, it is unclear whether or not CM predicts a general rejection of science (Landrum and Olshansky, 2019). Conspiracy believers might not reject science *per se*, but could just be highly selective in the science they trust (i.e. cherry-picking evidence corroborating one’s beliefs). In other words, mixed findings on the relation of CM and science skepticism might be explained by CM-enhancing motivated science reception, increasing trust in belief-*confirming* evidence, while decreasing trust in belief-*disconfirming* evidence at the same time (cf. Miller et al., 2016; Van Prooijen et al., 2020).

## Victim sensitivity

VS describes the tendency to hyper-vigilantly perceive and strongly respond to cues of untrustworthiness and mean intentions out of a fear of being duped by others (i.e. to become a victim; Baumert and Schmitt, 2016; Gollwitzer et al., 2013). VS is especially relevant in uncertain and complex situations (Gollwitzer et al., 2013) and has been associated with suspiciousness, paranoia, and general distrust of others (Schmitt et al., 2005), as well as a high need for control (Baumert and Schmitt, 2016). To our knowledge, there is no research so far that investigates VS in the context of science reception. However, it is plausible that individuals high in VS would feel particularly threatened and suspicious toward scientific evidence that is incongruent with their own beliefs, thereby enhancing their inclination toward motivated rejection of science.

## The present research

In two studies, we now examine whether personality traits, particularly CM and VS, modulate motivated science reception. In Study 1, we test the assumption that these two traits amplify the interaction of preexisting attitudes and a certain evidence position (i.e. the motivated science reception effect) on perceptions of researchers’ trustworthiness and the credibility of communicated findings. In Study 2, we test the same assumption and further extend our scope by further exploring broader personality traits, namely dispositional mistrust and intolerance for ambiguity. Both studies were preregistered (Study 1: https://osf.io/4f6pm, Study 2: https://osf.io/smcrp)^1^ and we openly share all study materials, anonymized data, analysis scripts, and supplemental tables (i.e. the supplemental material: https://osf.io/rwbzp/). Tables 1 (Study 1) and 2 (Study 2) report all means, standard deviations, correlations, and internal scale consistencies for the respective key variables.

**Table 1.** Means, standard deviations, correlations with confidence intervals, and internal consistencies (Study 1).

Variable	*M*	*SD*	1	2	3	4	5	6	7	8	9
1. Conspiracy mentality	2.82	1.00	*.88*								
2. Victim sensitivity	4.03	1.00	.11* [.00, .21]	*.90*							
3. Researchers’ trustworthiness	4.13	1.11	−.03 [−.13, .08]	−.06 [−.16, .04]	*.97*						
4. Credibility of findings	3.24	1.26	−.01 [−.12, .09]	−.10 [−.20, .00]	.76** [.72, .80]	*.89*					
5. Attitudes women’s quota	3.66	1.20	−.10 [−.20, .01]	.17** [.07, .27]	−.10 [−.20, .00]	−.04 [−.14, .06]	*.96*				
6. General trust in science	4.82	0.73	−.50** [−.57, −.42]	.13* [.02, .22]	.21** [.11, .30]	.11* [.00, .21]	.18** [.08, .28]	*.83*			
7. GES frequency	3.30	0.70	−.19**[−.28, −.09]	−.01 [−.11, .09]	.01 [−.09, .11]	−.02 [−.12, .08]	.10 [−.00, .20]	.22**[.12, .31]	*.75*		
8. GES experiences	6.17	3.34	−.15** [−.25, −.05]	.08 [−.02, .18]	−.05 [−.15, .05]	−.08 [−.18, .02]	.08 [−.02, .18]	.21** [.11, .30]	.54** [.46, .61]		
9. Science as powerful elite	3.88	0.79	.18** [.08, .28]	−.02 [−.13, .08]	.00 [−.10, .11]	.02 [−.08, .13]	.02 [−.08, .13]	−.12* [−.22, −.02]	.02 [−.08, .13]	−.06 [−.16, .04]	*.71*

Italicized values in the diagonal represent internal scale consistencies (Cronbach’s alpha). Values in square brackets indicate the 95% confidence interval for each correlation. *M* = mean; *SD* = standard deviation; GES = general engagement with science.

* *p* < .05. **p < .01.

## 1. Study 1

### Method

We conducted an online, between-subjects experiment, using an alleged news article about a scientific study about the consequences of implementing a women’s quota. Participants were randomly allocated to one of two article versions: the text describing the study was identical except for the last two sentences summarizing the researchers’ conclusions which were either in favor of or against benefits of the women’s quota. In the pro (vs con) condition, the article concluded “Due to the women’s quota, women perceive less [more] pressure to achieve the demanded performance and perceive the quota as a big relief [burden]. Our results show: the quota means less [more] stress for women.”

#### Sample

We determined our sample size by an a priori power analysis based on a question which is not the focus of the present article (see preregistration), arriving at a minimum target sample of 380 participants (anticipating some exclusions). We collected data from 382 participants. As preregistered, we excluded 12 participants who stated not to use their data which left us with a sample of *N* = 370 (69% female; 17–63 years old, *M* = 33.61, *SD* = 15.47, 80% with university degree or currently enrolled).

#### Procedure

First, we obtained informed consent and assessed demographics (age, gender, occupational status, academic discipline). Next, we measured participants’ VS with 10 items (e.g. “I cannot easily bear it when others profit unilaterally from me,” Schmitt et al., 2010), and, following, their CM with 8 items (including all 5 items from the original CM scale, e.g. “I think many very important things happen in the world, which the public is never informed about,” Bruder et al., 2013), and additionally 3 items adapted from Brotherton et al. (2013), to more specifically capture the science context (e.g. “A lot of scientific results are deliberately concealed from the public”). If not stated otherwise, all items in this study were assessed on scales ranging from 1 to 6.

Then, we randomly allocated participants to either the pro or con version of the alleged news article describing the study regarding the women’s quota (see above). Following, participants had to complete an attention check in which they had to select the correct statement about the article’s content out of four options. If they responded incorrectly, they had to read the article again. After that, participants responded to 14 bipolar adjective pairs assessing their perception of the researchers’ epistemic trustworthiness (e.g. incompetent–competent, insincere–sincere; Hendriks et al., 2015) and to six items assessing the credibility they ascribed to the communicated findings (e.g. “I think the research results are credible,” adapted from Altenmüller et al. (2023)). Next, we applied a manipulation comprehension check in which participants had to indicate the described conclusion on the study they read about (pro vs con quota) and measured their own attitudes toward the women’s quota using 13 items (adapted and extended from Gidengil (1996) and Mölders et al. (2018); e.g. “I support an obligatory women’s quota in organizations” and “A women’s quota leads to more justice”).

Finally, we assessed participants’ general trust in science (five items adapted from Wissenschaft im Dialog/Kantar (2022), e.g. “I trust in science and research”) and their perceptions of science as a powerful elite (five items, e.g. “Scientists are part of a powerful elite”). Furthermore, we asked about their general engagement with science (multiple-choice index of 15 possible engagement experiences and five items measuring engagement frequency on a scale from 1 = “never” to 5 = “almost daily”; Altenmüller et al., 2023; BBVA Foundation, 2011) and how they typically consume news (via newspapers, TV, radio, Internet, magazines, other). Finally, we asked whether we could use their data (“use-me” item: yes, no). Concluding, they could sign up for course credit or a voucher raffle (2× €50) and we thanked and debriefed them.

### Results

#### Motivated science reception and CM

**Figure 1.**
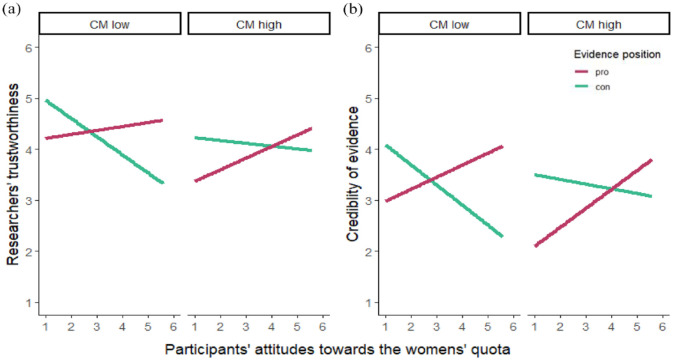
Display of motivated science reception (i.e. the interaction effect of participants’ attitudes and the presented evidence position) by CM in Study 1 for (a) perceptions of researchers’ trustworthiness and (b) the credibility of their findings. Simple slopes displayed for low CM (=2) and high CM (=5). CM = conspiracy mentality. Please see online version for color figure.

We conducted two multiple linear regressions with the predictor condition (con = −1, pro = 1), attitudes toward the women’s quota (mean-centered), CM (mean-centered), and their two-way and three-way interactions. While we found the typical motivated science reception effect (i.e. the condition × attitudes interaction) for both researchers’ perceived trustworthiness (*B* = 0.20, *SE* = 0.05, *p* < .001, *sr*² = .04) and credibility ascribed to their findings (*B* = 0.29, *SE* = 0.05, *p* < .001, *sr*² = .08), we did not find any effects of CM and its interactions in the two models (all: *p* > .054). Importantly, the three-way interaction between the condition, attitudes, and CM was not significant for trustworthiness (*B* = −0.03, *SE* = 0.04, *p* = .570, *sr*² < .01) or credibility (*B* = −0.03, *SE* = 0.05, *p* = .574, *sr*² < .01). This suggests that CM has no impact on motivated science reception (see Figure 1). Detailed regression results are displayed in Table S1 in the supplemental material.

#### Motivated science reception and VS

**Figure 2.**
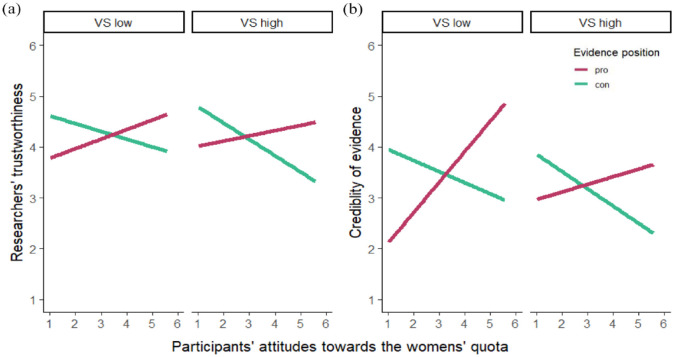
Display of motivated science reception (i.e. the interaction effect of participants’ attitudes and the presented evidence position) by VS in Study 1 for (a) perceptions of researchers’ trustworthiness and (b) the credibility of their findings. Simple slopes displayed for low VS (=2) and high VS (=5). VS = victim sensitivity. Please see online version for color figure.

We ran the two regression models again with VS (instead of CM), revealing the same pattern. As before, we found the expected motivated science reception effects with very similar size (both: *p* < .001), but the three-way (condition × attitudes × VS) interaction effect was not significant for trustworthiness (*B* = 0.01, *SE* = 0.05, *p* = .762, *sr*² < .01) or credibility (*B* = −0.05, *SE* = 0.05, *p* = .270, *sr*² < .01). This suggests that VS also has no impact on motivated science reception (see Figure 2). Interestingly, we found a significant effect of VS on credibility (*B* = −0.13, *SE* = 0.06, *p* = .047, *sr*² = .01), but all other (interaction) effects including VS were non-significant in both models (all: *p* > .056). Detailed regression results are displayed in Table S2 in the supplemental material.

**Table 2.** Means, standard deviations, correlations with confidence intervals, and internal consistencies (Study 2).

Variable	*M*	*SD*	1	2	3	4	5	6	7	8	9	10
1. Conspiracy mentality	2.77	1.07	*.90*									
2. Victim sensitivity	4.17	0.83	.17** [.07, .27]	*.85*								
3. Researchers’ trustworthiness	4.55	0.86	−.22** [−.31, −.12]	.08 [−.03, .18]	*.95*							
4. Credibility of findings	3.92	1.16	−.17** [−.27, −.07]	.07 [−.03, .17]	.73** [.67, .77]	*.88*						
5. Attitudes flu vaccine	4.74	0.80	−.52** [−.60, −.44]	−.04 [−.15, .07]	.23** [.13, .33]	.16** [.05, .27]	*.78*					
6. General trust in science	4.96	0.67	−.52** [−.59, −.44]	.05 [−.06, .15]	.33** [.23, .42]	.25**[.15, .34]	.51** [.42, .59]	*.79*				
7. GES frequency	3.11	0.72	−.26** [−.36, −.17]	−.10 [−.20, .00]	.13* [.03, .23]	.06 [−.04, .16]	.24**[.13, .34]	.31** [.22, .40]	*.74*			
8. Science as powerful elite	3.73	0.79	.23** [.13, .32]	−.01 [−.11, .09]	−.04 [−.14, .06]	−.08 [−.18, .02]	−.07 [−.18, .04]	−.07 [−.17, .04]	−.07 [−.17, .03]	*.63*		
9. Dispositional mistrust	2.93	1.07	.24** [.14, .33]	.18** [.08, .27]	−.13* [−.23, −.03]	−.12* [−.22, −.01]	−.12* [−.22, −.01]	−.23** [−.33, −.14]	.01 [−.10, .11]	.04 [−.07, .14]	*.84*	
10. Ambiguity intolerance	3.63	0.94	.11* [.01, .21]	.33** [.24, .42]	−.06 [−.16, .04]	−.06 [−.16, .04]	−.08 [−.18, .03]	−.09 [−.19, .02]	−.27** [−.36, −.17]	.04 [−.06, .14]	.13* [.03, .23]	*.71*

Italicized values in the diagonal represent internal scale consistencies (Cronbach’s alpha). Values in square brackets indicate the 95% confidence interval for each correlation. *M* = mean; *SD* = standard deviation; GES = general engagement with science.

* *p* < .05. **p < .01.

## 2. Interim discussion

This first study suggests that individuals’ CM and VS do not modulate their tendency to receive science in a way that fits to their preexisting attitudes. Noteworthily, VS correlated positively with general trust in science (Table 1), yet emerged as negative predictor for the credibility of the specific findings in the regression model, suggesting a complex interplay of VS, other variables, and situational characteristics in science reception. CM had a strong negative association with general trust in science (Table 1), but was not relevant for how the specific, presented scientific evidence was perceived. While we intentionally chose the polarizing issue of the women’s quota, maybe this topic did not feel relevant, consequential, or threating enough to tap into the latent suspicions some people might harbor about scientific research. Importantly, CM and VS remained non-significant moderators of motivated science reception when controlling for participants’ gender. We thus used a different topic in Study 2 which is likely associated with more suspiciousness: vaccinations.

Moreover, VS and CM might be considered rather narrow traits. CM is mostly used in a conspiracy-related context and VS is typically investigated in a justice-related context. While both of these areas can certainly overlap with science (e.g. research with implications for social justice issues and science-related conspiracies), they might only be relevant in specific situations. Thus, we designed our second study in a way that would more likely trigger participants’ latent suspiciousness and sense of injustice, creating a situation in which CM and VS should show effects on motivated science reception—if they have any. However, we also extended the scope of our examination to broader traits which might act as amplifying moderators of motivated science reception, namely dispositional mistrust and intolerance of ambiguity.

## 3. Study 2

### Method

Study 2’s method was very similar to Study 1. We used an alleged article about the effectiveness of a compulsory flu vaccination at universities. To heighten the potential for suspiciousness and feelings of injustice, the described study focused on the effect of mandatory vaccinations for only young people, which, in this context, explicitly meant students, not staff. Participants (only current students) were again randomly allocated to one of two article versions: The scientists’ conclusions were either in favor of or against the effectiveness of this vaccination policy. In the pro (vs con) condition, the article concluded:

Their investigation clearly shows that flu vaccinations of all adults younger than 26—this means, mostly students, not staff—will be a very effective [ineffective] policy to relieve hospitals and avoid shortages among university staff. The research group thus suggests, to consider implementing compulsory flu vaccinations for all people from 18 to 26 [keeping flu vaccinations voluntary].

#### Sample

We collected data from 446 people (our minimum target sample was 424 participants based on an a priori power analysis regarding a research question not relevant for the present article, see preregistration). As preregistered, we excluded 11 participants who stated not to use their data, 6 participants who completed the questionnaire implausibly fast (<180 seconds), and 56 participants who indicated another occupation than being a currently enrolled university student. This left us with a sample of *N* = 373 (81% female; 17–63 years old, *M* = 22.75, *SD* = 4.95).

#### Procedure

After obtaining informed consent, we measured attitudes toward flu vaccinations (eight items, e.g. “Healthy people do not need to get the flu vaccine,” adapted from Bonnevie et al. (2020) and Martin and Petrie (2017)).^2^ Next, we presented the alleged article (pro or con, see above), applied an attention check (as before, three options), and assessed participants’ perceptions of the researchers’ trustworthiness (as before) and the credibility of the findings (four items, slightly adapted from Study 1). Then, we measured VS and CM as before (except, we exchanged one of the additional items adapted from Brotherton et al. (2013) to better fit the context: “Some researchers intentionally fuel fears of future risks, because it is in their interest to do so”). Next, for purposes not relevant to the present article, we measured need for control with six items (e.g. “I like to have control over my own life,” based on Burger and Cooper (1979)) and perceived personal and societal control (six items, e.g. “I have control over my own life,” based on Greenaway et al. (2013), Paulhus and Van Selst (1990) and Pearlin and Schooler (1978)). We then assessed intolerance of ambiguity (e.g. “I dislike ambiguous situations,” based on McLain (2009)) and dispositional mistrust (e.g. “I tend to mistrust others,” adapted from Frazier et al. (2013)). Finally, we asked participants about their general engagement with science (only frequency, see Study 1), their demographics (age, gender, occupational status, academic discipline), as well as their political orientation. We concluded with the “use-me” item and the opportunity to sign up for a raffle (3× €50) or course credit, and thanked and debriefed the participants.

### Results

#### CM and VS

We ran the same four regression models as in Study 1, replicating the pattern of results (see Tables S3 and S4 in the Supplemental material). On both trustworthiness and credibility, we again observed the expected motivated science reception effect (i.e. the condition × attitudes interaction; all: *p* < .023). We did not find any effects of CM and its interactions (all: *p* > .083) or VS and its interactions (all: *p* > .126) in the respective models. Specifically, the three-way interaction between the condition, attitudes, and CM was again neither significant for trustworthiness (*B* = −0.01, *SE* = 0.05, *p* = .841, *sr*² < .01) nor for credibility (*B* = −0.08, *SE* = 0.07, *p* = .234, *sr*² < .01). Similarly, the three-way interaction between the condition, attitudes, and VS was also not significant for trustworthiness (*B* = −0.03, *SE* = 0.07, *p* = .620, *sr*² < .01) or credibility (*B* = 0.08, *SE* = 0.09, *p* = .405, *sr*² < .01).

#### Further traits

We then applied the same analytical strategy to explore whether dispositional mistrust or intolerance of ambiguity moderated the motivated science reception effect. However, none of these variables had any direct or two-way interaction effects on trustworthiness and credibility (mistrust, all: *p* > .102; ambiguity intolerance, all: *p* > .051). Crucially, the three-way interaction effects were also not significant (condition × attitudes × mistrust, both: *p* > .259; condition × attitudes × ambiguity intolerance, both: *p* > .109). Tables S5 and S6 in the supplemental material display the full regression results for each analysis.^3^

## 4. General discussion

In two studies, we investigated how dispositional differences might impact people’s tendency to perceive science in a motivated manner—that is, in line with their preexisting attitudes toward the research topic. As motivated science reception is likely driven by feelings of threat and more pronounced under perceived uncertainty and ambiguity, we focused on two specific traits, CM and VS, and two broader traits, dispositional mistrust and intolerance of ambiguity, which seem relevant in this context. Yet, none of these variables modulated the motivated science reception effect.

These results once again demonstrate the pervasive and persistent nature of motivated science reception (e.g. Altenmüller et al., 2021; Bender et al., 2016; Greitemeyer, 2014; Hutmacher et al., 2022; Nauroth et al., 2015, 2017; Nisbet et al., 2015; Rothmund et al., 2015, 2017). It is remarkable that traits so closely related to the presumed core drivers of motivated science reception (i.e. threat and uncertainty) have no tangible impact on it. Everyone seems to be similarly at risk of this bias, even individuals who tolerate ambiguity comparatively well and are not inclined toward feelings of threat and suspicion. This emphasizes the ubiquitous challenge motivated science reception poses to the effective dissemination of scientific knowledge.

Noteworthily, CM and dispositional mistrust showed considerable negative associations with general trust in science and trustworthiness and credibility in the specific vaccine-related research scenario (Study 2). However, CM did not correlate with specific trust variables in the women’s quota research example (Study 1). This demonstrates that CM is not necessarily associated with distrust in scientific evidence *per se*, but likely topic-, outcome-, and source-dependent (see also Landrum and Olshansky, 2019). Of note, in both of our studies, participants displayed rather low levels of CM (Study 1: *M* = 2.82, *SD* = 1.00; Study 2: *M* = 2.77, *SD* = 1.07; both on a scale from 1 to 6) which could have impaired our precision for estimating effects of high CM individuals.

The present article is, to the best of our knowledge, the first to investigate VS in the context of science reception. Beyond its well-documented relevance in the social justice area (e.g. Baumert and Schmitt, 2016; Gollwitzer et al., 2013; Schmitt et al., 2010), we now show that VS might also have small (and rather complex) effects on trust in science. For example, in Study 1, there was a small positive relation between VS and general trust in science (Study 1), but in the regression on evidence credibility, VS emerged as negative predictor. Thus, there might also be topic-specific effects at play. In general, these results warrant further investigation of VS in the context of science reception.

Overall, our results seem to suggest that personality might be irrelevant to motivated science reception. Of course, from the extensive personality space, we here only focused on two rather narrow traits and explored two further traits. Corroborating our insights, however, another research team very recently reported no modulating effects of the “dark factor” of personality (i.e. the common core of traits such as Machiavellianism, egoism, and psychopathy) in motivated reasoning about climate change (Hutmacher et al., 2024). While all these traits can be considered to be potential *amplifiers* of motivated science reception, one could also take a different approach. Some dispositional differences such as traits associated with openness or perspective taking might *reduce* the motivated rejection of science which is incongruent to one’s beliefs. For example, intellectual humility has been identified as alleviating factor to polarization (e.g. Bowes et al., 2022; Krumrei-Mancuso and Newman, 2020; Leary et al., 2017; Porter and Schumann, 2018). Thus, future research might be able to identify other dispositions that are relevant to motivated science reception, after all.

## 5. Conclusion

Motivated science reception is a pervasive and persistent phenomenon. Across two studies, a range of theoretically relevant personality traits (VS, CM, dispositional mistrust, intolerance of ambiguity) did not modulate the strength of this effect. Future research is needed to get a more complete picture of whether and how personality might impact such biased science reception. While it is scientifically interesting to understand the theoretical and empirical connection between personality and (motivated) science reception, it also has crucial practical relevance. Knowing who is especially at risk of rejecting scientific evidence (or blindly believing in pseudo-scientific claims) in a motivated manner might be important for tailoring effective science communication.

## Notes

1. Only Hypothesis 2 in the preregistration of Study 1 and Hypotheses 2 and 3 in the preregistration of Study 2 are relevant for the present article.

2. In Study 2, we randomized whether participants responded to the attitude measure before or after reading the stimulus and responding to the trustworthiness and credibility measures. Due to an interference with the internal feedback loops of the attention check, this led to a programming error resulting in missing attitude data for *n* = 53 participants.

3. Exploratorily, we also ran the analyses with the disposition *need for control*, as one could make the case that individuals satisfy their need for control by engaging in motivated reasoning to reduce perceived uncertainty elicited by incongruent information threatening their sense of control. Need for control had no significant direct or interaction effects in the models. We report the results in the Supplemental material (Table S7).
